# Long-Term Bidirectional Global Health Workforce Exchange: Coordination, Continuity, and Financing

**DOI:** 10.5334/aogh.5515

**Published:** 2026-11-13

**Authors:** Kinuko Nagayoshi, Kohei Nakata, Junko Yoshizumi, Shiho Tajiri, Shuji Shimizu, Tomohiko Moriyama

**Affiliations:** 1 Overseas Exchange Center, International Medical Department, Kyushu University Hospital, Fukuoka, Japan; 2 Telemedicine Development Center of Asia, International Medical Department, Kyushu University Hospital, Fukuoka, Japan; 3 Kyushu University, Fukuoka, Japan

**Keywords:** global health workforce, bidirectional exchange, international health professions education, partnership continuity, financing, university hospital

## Abstract

*Background:* International health workforce exchanges are widely used to support training and institutional collaboration, yet many remain dependent on individual relationships, short-term funding, and episodic activities. Evidence regarding the organizational structures, continuity measures, and financing arrangements required for long-term bidirectional exchange remains limited.

*Objective:* To describe the long-term implementation of a bidirectional international health workforce exchange platform and examine its organizational coordination, partnership continuity, financing evolution, and formal institutional development.

*Methods:* We conducted a descriptive implementation study (April 2015–March 2026) on a bidirectional exchange platform coordinated at Kyushu University Hospital in Japan. Inbound training included 587 trainee participations from 54 countries. Outbound outreach visits were summarized per annual trends, professional composition, destinations, and financing mix (internal university funds, program-based external funding, and other/mixed sources). Continuity was quantified using institution- and country-level repeat indicators. Formal partnership development was assessed using memoranda of understanding (MoUs).

*Findings:* Institution-level continuity was substantial: 31.1% of sending institutions contributed ≥2 inbound trainee participations (100/322, 95% CI: 26.3%–36.3%) and 27.6% contributed trainee participations in ≥2 calendar years (89/322, 95% CI: 23.0%–32.8%); repeat intensity showed a long-tail pattern (≥10 inbound trainee participations in 5.3%). Country-level continuity was high (country repeat rate: 72.2% [39/54, 95% CI: 59.1%–82.4%]; multiyear country repeat rate: 70.4% [38/54, 95% CI: 57.2%–80.9%]). Outbound activities comprised 109 visits involving 405 participants, 39 countries, and 139 facilities, with diversification in professional composition and destinations over time. Financing evolved from internal expansion to increased external funding following the COVID-19 disruption. Eight platform-related MoUs were concluded.

*Conclusions:* Centralized coordination, routine continuity monitoring, and progressive financing diversification may contribute to the long-term institutionalization of bidirectional international health workforce exchange in university hospital settings.

## Introduction

International health workforce development, encompassing short-term observerships, structured training, and outbound outreach (capacity-building visits), has become a key approach to cross-border health professions education and institutional collaboration [1]. Despite this, many exchange activities remain ad hoc, driven by individual relationships with limited institutional support. Ethical and operational concerns have been raised about short-term global health training experiences [2]. Therefore, bidirectional exchange has been emphasized as a core principle of equitable and sustainable global health partnerships [3]. Host institutions also report practical burdens and expectations regarding short-term learners, underscoring the need for organized coordination rather than fragmented arrangements [4, 5]. Consequently, health system leaders and policymakers have limited evidence on how to operationalize sustainable exchange platforms, how to finance them over time, and how to quantify whether partnerships persist rather than remain episodic [6].

We examined the long-term implementation of a bidirectional exchange platform at a university hospital in Japan, coordinated through a standardized hub that integrates inbound training and outbound outreach across multiple disciplines and professions.

This study aimed to examine the platform across four implementation domains: (i) organizational structure and coordination, (ii) workforce exchange scale and reach, (iii) continuity of institutional and country-level partnerships, and (iv) financing evolution and formal partnership development, including memoranda of understanding (MoUs).

## Methods

### Study design and setting

We conducted a descriptive implementation study of a bidirectional international exchange platform coordinated by the Overseas Exchange Center (OVEX), Kyushu University Hospital. This study used the routine program registries and related administrative records. Inbound training and outbound outreach were analyzed from April 2015 to March 2026 (FY2015 to FY2025). The inbound registry recorded cumulative trainee participations rather than confirmed unique individuals; therefore, repeated participation by the same individual could not be systematically excluded. Inbound training duration varied by program and specialty but was most commonly approximately 1–3 months.

### Organizational structure and coordination model

The exchange platform was organized around a centralized coordination model within Kyushu University Hospital. OVEX served as the operational hub for both inbound and outbound activities across multiple clinical departments and professional groups. Its functions included coordination of applications, interdepartmental scheduling, institutional procedures, communication with partner institutions, and logistical support for international activities (Supplementary Figure S1). Other hospital-based units provided complementary support for remote education, technical assistance, and international patient services, but the coordination of workforce exchange activities was centralized through OVEX. Funding and resource allocation were determined within individual internal or externally funded programs, whereas OVEX coordinated institutional procedures, scheduling, communication, and cross-departmental implementation. The present administrative dataset did not systematically capture broader governance processes such as formal priority-setting or shared decision-making with partner institutions.

### Data sources

We analyzed (1) an inbound registry of trainee counts according to year, country, and sending institution (aggregated; no individual identifiers in analytic outputs); (2) an outbound trip-level registry (date, destination, number of participants, professional composition, and funding source); (3) funding/project records; and (4) an MoU registry (partner, date, agreement level, and agreements in progress).

### Funding source classification

Outbound activities were categorized into five funding-related groups: (1) university/hospital internal funds; (2) Japanese government program funding for the international deployment of medical technologies; (3) Japanese government development cooperation (ODA) program funding; (4) research grants; and (5) other external funding (including education partnership programs, industry contracts, and other minor sources). Table 1 summarizes the major externally funded programs, defined as fiscal-year-based projects supporting bilateral activities (inbound and/or outbound), whereas the outbound financing mix reflects all outbound activities, including smaller funding sources not itemized in Table 1.

**Table 1:** Summary of major externally funded projects supporting the bidirectional platform.

FUNDING CATEGORY	PROGRAM TYPE	CLINICAL AREA	TARGET REGION/COUNTRIES (EXAMPLES)	IMPLEMENTATION PERIOD	NO. OF PROJECTS
Japanese government program for international deployment of medical technologies	Capacity building (inbound/outbound)	Gastroenterology / Endoscopy / Gastrointestinal surgery	Philippines, Mongolia, Kenya, Bhutan, Lao People’s Democratic Republic, Myanmar	2019–2025	7
Japanese government development-cooperation program	Knowledge co-creation / training	Endoscopy / Colorectal surgery	India, Central Asia and Caucasus; Nikkei communities in Latin America	2018–2025	4
Japanese government education-partnership program	Human resource development (On-the-job training)	Endoscopy / Surgery	Egypt	2019–2025	2
National research grant	Remote medical education (hybrid/remote)	Remote medical education	Latin America (e.g., Brazil, Colombia, Peru, etc.)	2016–2023	2
Other foundation	Remote medical education Train-the-trainer	Telemedicine engineering	Asia	2015–2023	2
Some projects receive support multiple times over several fiscal years.					

### Outcomes and indicators

Bidirectionality was operationally defined at the platform level as the coexistence of inbound training and outbound collaborative activities within the same institutional platform and did not require reciprocal exchange with every partner institution. Institution-level bilateral exchange was defined as the occurrence of both inbound and outbound activities involving the same partner institution. Institution names were harmonized for minor spelling and naming variations before matching records across the inbound and outbound registries. Bidirectionality was not intended as a direct measure of equity, reciprocity, or mutual benefit. Program volume and reach were summarized for inbound training (total trainee participations, countries of origin for trainees, and number of countries) and outbound outreach (total visits, participants, and destination countries and facilities), with annual trends in volume, professional mix, and destinations. Partnership continuity for inbound training was quantified at institutional and country levels. We defined the institutional repeat rate as the proportion that sent trainees two or more in total (≥2 inbound trainee participations), and the multiyear repeat rate as the proportion that sent trainees two or more calendar years (≥2 calendar years); repeat intensity was summarized as 1, 2–3, 4–9, and ≥10 inbound trainee participations. At the country level, we similarly defined country and multiyear repeat rates. These measures should be interpreted as indicators of relational continuity rather than direct measures of educational effectiveness, equitable partnership, or downstream health-system impact. FY2020 and FY2021, during which no exchange activities occurred because of COVID-19–related travel restrictions, were retained within the overall observation period. Because institutional- and country-level repeat rates used sending institutions and countries as their respective denominators, these inactive years did not directly affect the repeat-rate denominators. For temporal trend analyses, FY2020 and FY2021 were retained as zero-activity years. Formal partnership development was assessed using the number and organizational level of concluded MoUs in which our bidirectional exchange platform played a role in at least one of the following: (i) initiating or coordinating inbound training and/or outbound outreach activities that led to the agreement, (ii) serving as administrative or operational contact during MoU preparation, or (iii) supporting implementation planning after signature. As an illustrative example of a platform-involved MoU, we coordinated bidirectional activities with Mongolia over multiple years with phased funding (internal support in 2018–2019 followed by external program funding from 2022 onward) and a formal MoU milestone (Supplementary Figure S2).

### Statistical analysis

Categorical continuity indicators (institutional and country-level repeat and multiyear repeat rates) are reported with 95% confidence intervals calculated using the Wilson score method. Temporal trends in annual country reach and annual trainee volume were assessed using linear regression and Spearman’s rank correlation; these analyses are exploratory and descriptive, given the limited number of annual observations. All analyses were performed using Python (SciPy).

### Ethics and data protection

The institutional ethics committee of Kyushu University Hospital determined that formal ethical review was not required because the study used only aggregated administrative program data with no identifiable individual information.

## Results

### Platform characteristics and overall reach

Between April 2015 and March 2026 (FY2015–FY2025), the OVEX coordinated a bidirectional platform comprising inbound training and outbound outreach. The inbound training included 587 trainee participations from 54 countries. The top five countries of origin for inbound trainee participations were Vietnam (42), the Philippines (41), Thailand (39), Mongolia (31), and China (28). Outbound outreach consisted of 109 visits involving 405 participants across 39 countries and 139 facilities. No inbound or outbound activities were conducted during 2020–2021 due to COVID-19–related travel restrictions. Institution-level bilateral exchange was documented for at least 75 partner institutions during the study period.

### Inbound workforce participation and geographic reach

Inbound training demonstrated temporal changes not only in overall volume, but also in professional composition and country reach (Figure 1A). Across the years, with active exchanges, trainee profiles were dominated by physicians, with additional participation from nurses, other medical professionals, technical engineers, and students. The number of partner countries varied over time, with a clear interruption during the COVID-19 period (no inbound activities in 2020–2021) followed by an increase in country reach in subsequent years. Across the study period, the annual number of partner countries increased significantly (linear trend, P = 0.012; Spearman’s ρ = 0.83, P = 0.006), rising from 16 countries in FY2015 to 31 in FY2025. In contrast, annual trainee volume did not show a significant temporal trend (linear trend, P = 0.44; Spearman’s ρ = 0.47, P = 0.21). Because no activities occurred during 2020–2021, the observed increase in country reach partly reflects recovery from the COVID-19 interruption rather than an independent secular trend and should be interpreted with this constraint in mind.

**Figure 1:**
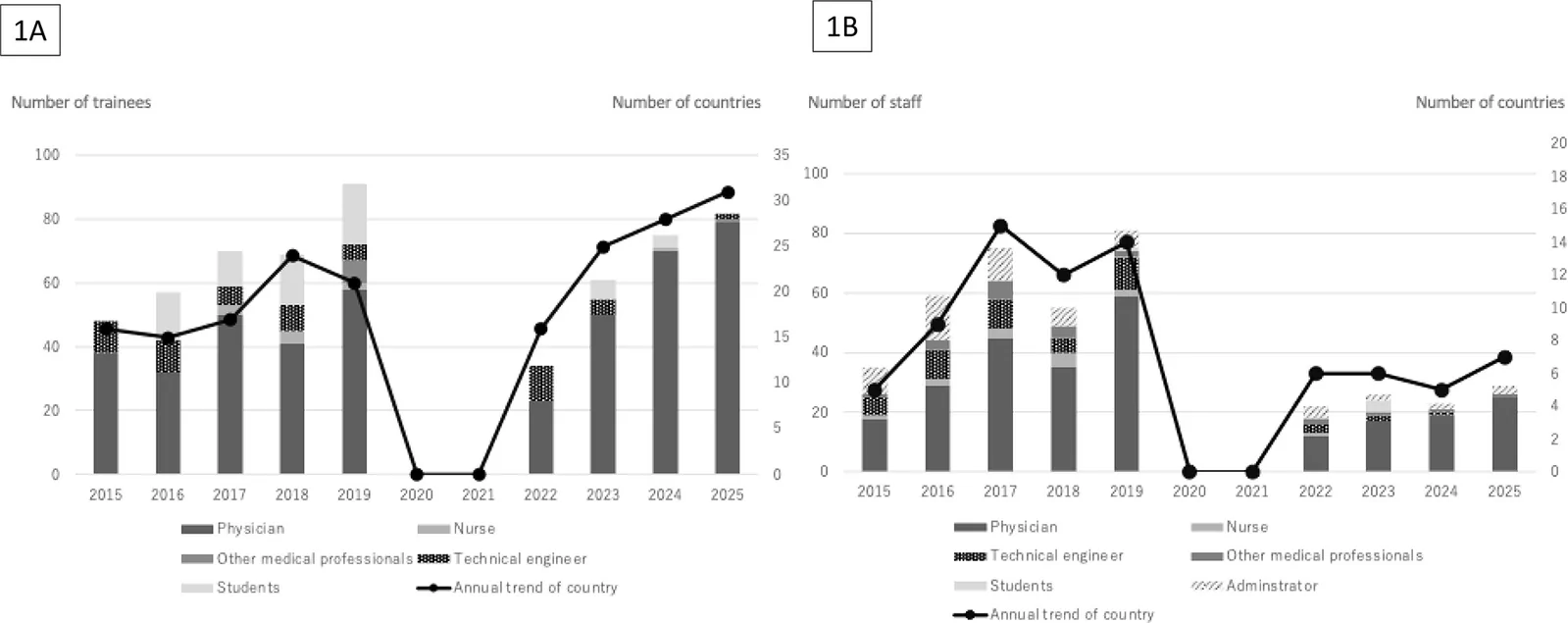
Annual trends in inbound trainee participations and outbound outreach participants coordinated by the bidirectional exchange platform. This figure presents the annual participant volumes for **(1A)** inbound and **(1B)** outbound activities, and the number of partner countries over time. No inbound or outbound activities were conducted during 2020–2021 due to COVID-19–related travel restrictions. Calendar year labels denote fiscal years (April–March).

### Institutional-level partnership continuity

The institution-level continuity was substantial. Among 322 sending institutions, 31.1% sent ≥2 inbound trainee participations (100/322, 95% CI: 26.3%–36.3%), and 27.6% sent trainee participations in ≥2 calendar years (89/322, 95% CI: 23.0%–32.8%).

Repeat engagement showed a long-tail pattern: 64.8% of institutions contributed trainee participations only once, whereas 21.5% sent 2–3 inbound trainee participations, 8.4% sent 4–9 inbound trainee participations, and 5.3% sent ≥10 inbound trainee participations.

### Country-level partnership continuity

Country-level continuity was also high. Across 54 countries with inbound activity, the country repeat rate (total ≥2) was 72.2% (39/54, 95% CI: 59.1%–82.4%) and the multiyear country repeat rate (nonzero years ≥2) was 70.4% (38/54, 95% CI: 57.2%–80.9%), indicating sustained engagement across multiple years for most partner countries.

### Financing of inbound exchange

Inbound financing shifted over time: the early years relied more on self-pay and internal resources (institutional funds and internal research budgets), whereas the post-COVID years showed increased reliance on external programs and third-party funding (Figure 2).

**Figure 2:**
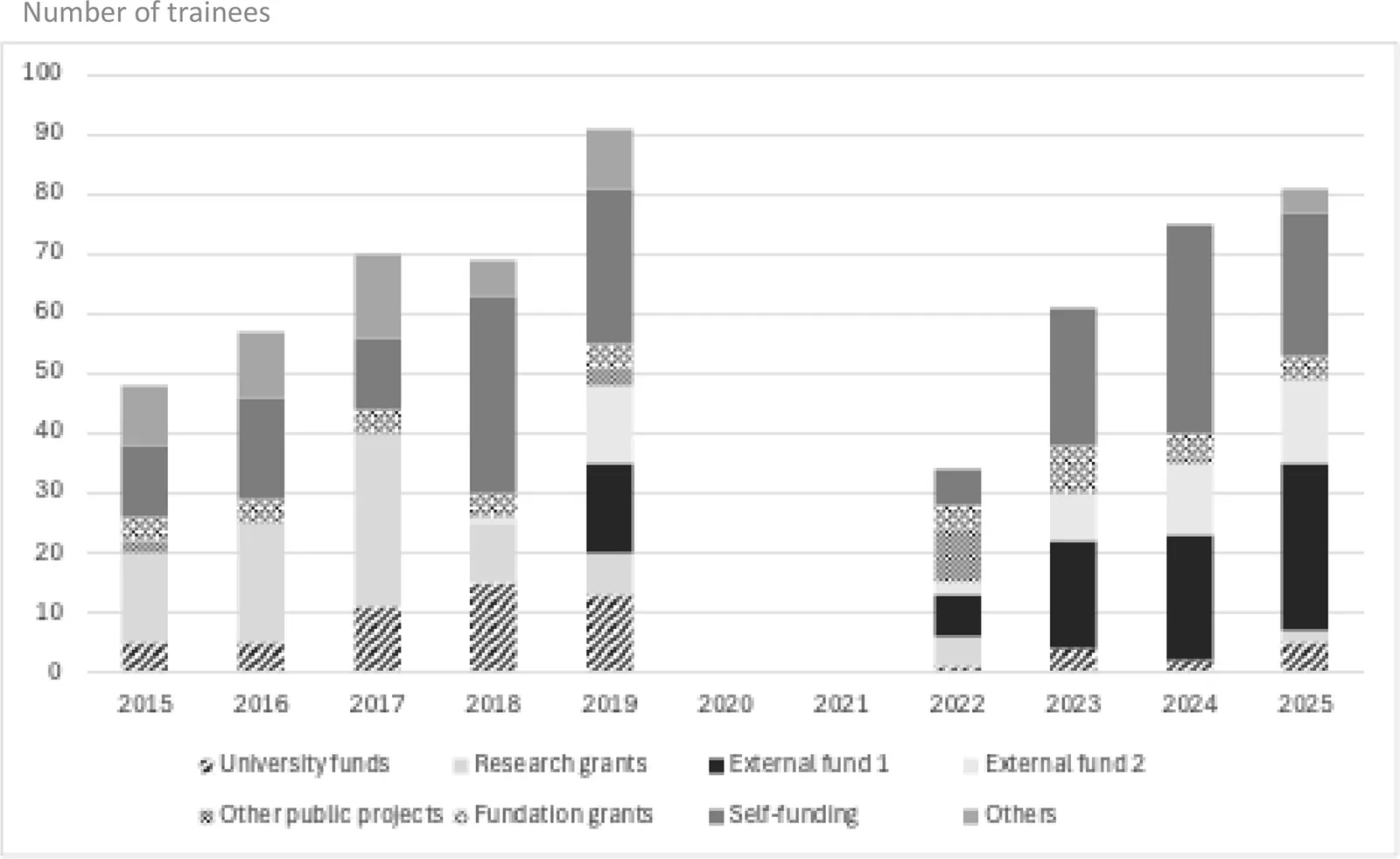
Annual financing mix of inbound trainee participations by funding source. The stacked bars show the annual distribution of inbound trainee participations across the funding categories. Funding sources were categorized as self-funding, university/hospital internal funds, Japanese government program funding for the international deployment of medical technologies (External Fund 1), Japanese government development-cooperation program funding (External Fund 2), research grants, other public projects, foundation grants, and other external funding. Calendar year labels denote fiscal years (April–March).

### Outbound workforce participation and geographic reach

Outbound outreach demonstrated temporal changes not only in volume, but also in professional composition and destination patterns (Figure 1B). Multi-professional participation, combining clinicians with nonclinical roles (e.g., engineers and administrative staff), was observed across multiple programs. Destination profiles have evolved over time, with concentrated engagement in several partner countries while maintaining a broader geographic reach.

### Financing of outbound activities

The annual financing mix for outbound outreach diversified over time (Figure 3), reflecting a transition from early expansion supported primarily by internal university funds to increased utilization of program-based external funding for international activities following the COVID-19 disruption period.

**Figure 3:**
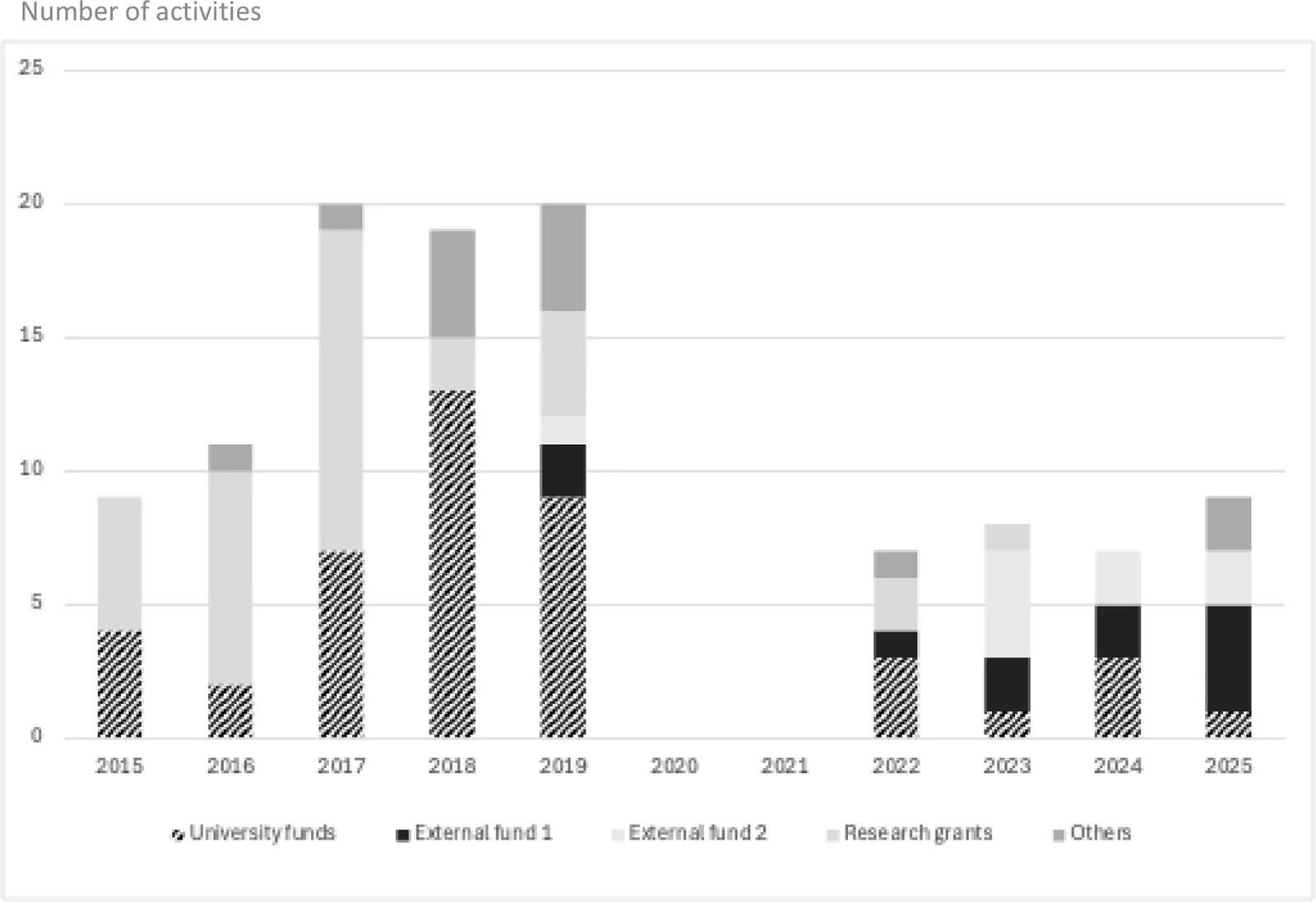
Annual financing mix of outbound outreach activities according to funding source. The stacked bars show the annual distribution of outbound activities across the funding categories. Notes: Funding sources were categorized as university/hospital internal funds, Japanese government program funding for the international deployment of medical technologies (external fund 1), Japanese government development-cooperation program funding (external fund 2), research grants, and other external funding sources. Calendar year labels denote fiscal years (April–March).

### Formal partnership development

Eight platform-involved MoUs were identified during the implementation period (three university, one hospital, and four department levels), with two additional agreements in progress. Concluded MoUs involved partner institutions from Indonesia (n = 2), Mongolia (n = 1), Thailand (n = 1), Chile (n = 2), Russia (n = 1), and Vietnam (n = 1), as summarized in Supplementary Table S1.

## Discussion

This longitudinal descriptive study examined the organizational coordination, continuity, financing, and formal partnership development of a bidirectional international health workforce platform operated for more than a decade. The principal contribution is not the scale of international exchange alone, but the identification of practical organizational features associated with continuity over time: centralized coordination, measurable continuity of institutional relationships, progressive diversification of financing sources, and formalization of selected partnerships through MoUs.

First, the partnership continuity is measurable and substantial. At the institution level, 31.1% of sending institutions sent ≥2 inbound trainee participations (100/322, 95% CI: 26.3%–36.3%) and 27.6% engaged across multiple calendar years (89/322, 95% CI: 23.0%–32.8%), with a long-tail pattern indicating a broad base of one-time partners alongside a smaller set of high-intensity sending institutions. At the country level, the country repeat rate was 72.2% (39/54, 95% CI: 59.1%–82.4%), and the multiyear country repeat rate was 70.4% (38/54, 95% CI: 57.2%–80.9%). These pragmatic continuity indicators provide a transferable way to quantify whether exchange programs function as sustained partnerships rather than episodic events, which has often been unclear in prior reports [6–8]. Similar international visiting scholar/observership programs have been reported, but continuity and institutionalization metrics have not been consistently presented [7]. For example, a multi-institutional Korean survey documented 1,496 international scholars from 80 countries across 8 surgical departments over a 5-year period, but did not report institutional- or country-level continuity indicators comparable to those presented here [7]. More broadly, a systematic review of 28 published international surgical training exchanges found that only one (3.6%) was structured bidirectionally, with the large majority representing one-directional, North-to-South training flows [8]. Few previous reports have quantified the continuity of international exchange programs. In our study, repeat engagement could be assessed at both institutional and country levels, allowing sustained relationships to be distinguished from one-time exchanges.

Second, the platform matured from an early phase, in which exchange activities were expanded primarily through internal university funding, to a later phase, characterized by the increasing acquisition and utilization of program-based external funding. In our setting, internal funding was initially used to establish and disseminate the core tele-education infrastructure and operational workflows, and the subsequent scale-up of international exchange activities was supported by external program funding. The annual financing mix (Figure 3) and portfolio of major external programs (Table 1) illustrate this staged trajectory, in which internal seed funding enabled early expansion and proof-of-concept, and diversification of external funding coincided with expansion of the program portfolio and reduced reliance on a single internal funding source [9]. The descriptive design does not establish that financing diversification itself caused program continuity or resilience; this pattern may be useful for institutions seeking to reduce dependence on a single internal funding source. The disruption of international mobility during the COVID-19 pandemic highlights the importance of building resilient, institution-led platforms rather than relying on ad hoc exchanges [10], and virtual/hybrid models illustrate how structured platforms can maintain collaboration when physical mobility is constrained [11].

Third, our bidirectional exchange platform functions as a standardized coordinating hub that may support more consistent delivery of bidirectional activities and reduce duplication of administrative tasks across frontline clinical departments. Central coordination allows routine workflows (e.g., scheduling, documentation, and stakeholder communication) to be handled consistently, enabling clinical units to participate without disproportionate administrative loads.

Formal partnership development provides a further, complementary indicator of platform maturation. We identified eight platform-involved MoUs (two in progress) across multiple countries and agreement levels. Rather than treating MoUs as evidence of institutionalization in themselves, the program conceptualizes them as one indicator of formal partnership development, alongside the operational continuity measures described above [12]. The Mongolia case (Supplementary Figure S2) illustrates how phased funding and bidirectional activities can align with MoU milestones, offering a concrete example of how operational exchanges may translate into formal partnership development.

Our findings suggest three practical considerations for hospitals and policymakers seeking to organize international exchange platforms. First, a centralized and standardized coordination hub may improve operational efficiency and protect frontline capacity, enabling wider clinical participation while reducing the administrative burden. Second, routine program monitoring could incorporate continuity indicators (repeat and multiyear repeat measures at the institutional and country levels), allowing hospitals to track partnerships over time with reproducible measures. Third, staged and diversified financing may be a pragmatic approach for institutions seeking to reduce dependence on a single funding source, although this descriptive study cannot establish that diversification itself improves resilience or scalability. Internal funding can seed early activity, while the deliberate acquisition of diversified external funding streams may help sustain and expand the platform across periods of disruption. The Fair Trade Learning framework provides a broader perspective for evaluating international educational partnerships by emphasizing reciprocity, community responsiveness, responsible use of resources, and sustained engagement [13]. Our measures of bidirectionality, continuity, and financing capture some operational dimensions relevant to these principles, particularly sustained engagement and resource stewardship. However, the present administrative dataset does not capture partner-defined priorities, perceived reciprocity, power sharing, or mutual benefit. Future evaluations should therefore incorporate partner perspectives and equity-oriented measures alongside the operational indicators reported here.

The strengths of the study include a long observation period, bidirectional scope, multidisciplinary participation, and the use of transparent continuity indicators that can be adopted by other institutions. This study also integrates the financing evolution and formal partnership development outcomes, which are often absent in program reports. The limitations include the descriptive design and reliance on operational registries, which may vary in completeness across years; this design does not permit causal inference between organizational features (e.g., centralized coordination, financing diversification) and program continuity. The continuity indicators are pragmatic measures of sustained engagement, but do not capture all dimensions of educational effectiveness or downstream patient-level outcomes. The study relied on administrative records from the coordinating institution and did not capture the perspectives of trainees or partner institutions; accordingly, it cannot assess educational outcomes, perceived mutual benefit, or equity within the partnerships. Repeat engagement and MoUs are proxy indicators of relational continuity and formalization; they do not demonstrate educational effectiveness, workforce retention, or improvements in patient care. Finally, all data derive from a single coordinating hub in Japan, which may limit generalizability to platforms with different organizational or funding structures.

## Conclusions

The bidirectional platform at Kyushu University Hospital coordinated and maintained international training and outbound outreach activities for more than a decade. Continuity was quantifiable at both the institutional and country levels, while financing shifted from reliance on internal resources toward a broader portfolio of external programs. Selected operational relationships were formalized through MoUs. These findings provide an operational model for institutions seeking to organize long-term international workforce exchange, while partner perspectives, educational outcomes, and health-system effects require further evaluation.

## Data Sharing and Accessibility

Data supporting the findings of this study are available from the corresponding author upon reasonable request. The data are not publicly available due to privacy and ethical considerations.

## Additional Files

The additional files for this article can be found as follows:

Supplementary Table S1. Memoranda of Understanding concluded during the implementation period.

Supplementary Figure S1. Schematic overview of OVEX’s standardized hub functions for inbound training and outbound outreach.

Supplementary Figure S2. Mongolia case example.
