# Supplementary material for: Long-Term Bidirectional Global Health Workforce Exchange: Coordination, Continuity, and Financing
Source: Ann Glob Health. 2026 Nov 13;92(1):114. doi: 10.5334/aogh.5515 (PMC13761882; doi:10.5334/aogh.5515)
Supplement: Supplementary file 1 [file agh-92-1-5515-s1.pdf]

Supplementary Table S1. Memoranda of Understanding concluded during the implementation period.

	Partner institution	Agreement level	Date (month/year)	Status
Indonesia	University of Brawijaya, Faculty of Medicine	University	Dec-2025	Concluded
Chile	Clinica Alemana	Hospital	Oct-2025	Concluded
Mongolia	Mongolian National University of Medical Sciences	University	Aug-2024	Concluded
Thailand	Chulalongkorn University, Faculty of Medicine	University	May-2024	Concluded
Indonesia	Brawijaya University	Department	Sep-2019	Concluded
Russia	Vladivostok Clinical Railway Hospital	Department	Jul-2018	Concluded
Chile	Clinica Alemana	Department	Jun-2018	Concluded
Vietnam	Center of Science and Technology Applications	Department	Jun-2018	Concluded
